# Detailed Report on 2014/15 Influenza Virus Characteristics, and Estimates on Influenza Virus Vaccine Effectiveness from Austria’s Sentinel Physician Surveillance Network

**DOI:** 10.1371/journal.pone.0149916

**Published:** 2016-03-14

**Authors:** Monika Redlberger-Fritz, Michael Kundi, Theresia Popow-Kraupp

**Affiliations:** 1 Department of Virology, Medical University Vienna, Vienna, Austria; 2 Institute of Environmental Health, Center for Public Health, Medical University Vienna, Vienna, Austria; Icahn School of Medicine at Mount Sinai, UNITED STATES

## Abstract

**Background:**

Influenza vaccine effectiveness (VE) is influenced by the antigenic similarity between vaccine- and circulating strains.

**Material and Methods:**

This paper presents data obtained by the Austrian sentinel surveillance system on the evolution of influenza viruses during the season 2014/15 and its impact on influenza vaccine effectiveness in primary care in Austria as estimated by a test-negative case control design. VE estimates were performed for each influenza virus type/subtype, stratified by underlying diseases and adjusted for age, sex and calendar week of infection.

**Results:**

Detailed genetic and antigenic analyses showed that circulating A(H3N2) viruses were genetically distinct from the 2014/15 A(H3N2) vaccine component indicating a profound vaccine mismatch. The Influenza A(H1N1)pdm09 viruses were antigenically conserved and matched the respective vaccine component. Influenza B viruses were lineage-matched B/Yamagata viruses with a clade-level variation. Consistent with substantial vaccine mismatch for the A(H3N2) viruses a crude overall VE of only 47% was estimated, whereas the VE estimates for A(H1N1)pdm09 were 84% and for influenza B viruses 70%. Increased VE estimates were obtained after stratification by underlying diseases and adjustment for the covariates sex and age, whereby the adjustment for the calendar week of infection was the covariate exerting the highest influence on adjusted VE estimates.

**Conclusion:**

In summary, VE data obtained in this study underscore the importance to perform VE estimates in the context of detailed characterization of the contributing viruses and also demonstrate that the calendar week of influenza virus infection is the most important confounder of VE estimates.

## Introduction

Influenza viruses constantly change through genetic and antigenic drift which causes a considerable problem for the production of effective vaccines [1, 2]. In order to assess the evolutionary changes of the circulating strains, a continuous virological surveillance is conducted globally. Based on these surveillance results influenza vaccine antigens are reviewed annually and revised as needed according to the most recent changes [3]. Due to the time required for the vaccine production process the statement concerning the vaccine strains has to be delivered months in advance of the actual influenza season. As a consequence of this an antigenic mismatch between the strains included in the vaccine and the circulating strains is not infrequently observed and limits the effectiveness of the vaccine [4–7]. Estimates of VE as low as 28% and even lower have been reported for seasons with profound vaccine mismatch [5, 8].

Up to now data on influenza VE in Austria, a country with a traditionally low influenza vaccine uptake [9], are not available. Influenza virus activity and evolution is monitored in Austria by the Diagnostic Influenza Network Austria (DINÖ), a network of sentinel physicians throughout the country which collect clinical samples as well as basic clinical information. In the 2014/15 season in Austria a considerable heterogeneity in the proportionate mix of circulating strains and their match to the vaccine components for this season was observed. Like in most European countries the 2014/15 influenza season was dominated by A(H3N2) viruses causing 56% of the laboratory confirmed infections. Phylogenetic analysis revealed the circulation of A(H3N2) viruses clustering with clade 3C.3b and 3C.2a viruses, indicating substantial vaccine mismatch. In addition to the mix of mismatching A(H3N2) strains, influenza A(H1N1)pdm09 and influenza B viruses with an agreeable match to the respective vaccine strains were detected to about equal parts in 44% of the circulating viruses.

In order to obtain reliable estimates of VE for Austria in the 2014/15 season basic clinical information from the Austrian sentinel surveillance system was linked with data of genetic and antigenic monitoring of influenza vaccine virus relatedness. The overall and component specific VE against medically attended, laboratory confirmed influenza infection was estimated using the test negative case control design. This methodology, first described for the 2004/05 influenza season in Canada [10, 11] has revolutionized VE monitoring and is now used by many countries for the rapid evaluation of influenza VE, during and at the end of the season [12–17]. Estimates of VE in Austria will be presented in the context of detailed genetic and antigenic analysis of the contributing sentinel viruses.

## Material and Methods

### Sentinel influenza surveillance system and samples tested

Sentinel surveillance for influenza viruses is conducted annually from October (calendar week 40) through April (week 16 of the following year). Sentinel physicians (general practitioners and paediatricians throughout Austria) forming part of the Diagnostic Influenza Network Austria (DINOE), provided with swab sample kits, collect nasopharyngeal swabs from patients presenting with influenza like illness as defined by the ECDC [18]. The samples are submitted to the NIC Austria located at the Department of Virology, Medical University Vienna for further analysis. Along with the sentinel samples information on age, gender, underlying health conditions (like diabetes, cardio-vascular diseases, chronic lung diseases, malignant diseases), adiposity, smoking habits, vaccination status, kind of vaccine (trivalent inactivated vaccine = TIV or live attenuated vaccine = LAIV), prior influenza immunisation for season 2013/14, date of onset of symptoms and of specimen collection was obtained.

#### Ethics statement

This study was conducted at the Department of Virology of the Medical University of Vienna, the WHO NIC for Austria, and is a retrospective analysis of viral and epidemiological data of fully anonymized material collected during the annual influenza surveillance within the frame of Austria’s Sentinel Physician Surveillance Network and therefore an informed consent could not be obtained. The study was performed according to the Declaration of Helsinki and its Ammendments and the research protocol was approved in its current form by the ethics committee of the Medical University of Vienna (EK: 1670/2015).

### Vaccination

In Austria influenza vaccination is usually carried out from the beginning of October to the end of November and the non-adjuvanted, inactivated trivalent influenza vaccine is primarily used. Children below 36 months of age receive half the amount of the adult’s vaccine dose, and people above 65 years of age are vaccinated with MF095 adjuvanted vaccines. Life attenuated influenza vaccine is approved for individuals 2 to 18 years old in Austria since 2014. TIV for the season 2014/15 contained the following recommended vaccine strains: A(H3N2): A/Texas/50/2012-like virus, A(H1N1)pdm09: A/California/7/2009-like virus and Influenza B: B/Massachusetts/2/2012-like virus.

### Laboratory testing

#### Influenza virus detection and genotyping

Sentinel specimens were tested for influenza viruses by reverse transcription realtime PCR (RT realtime PCR) as described previously [19]. After RNA extraction (using Nucli Sens–Easy Mag (BioMerieux, SA, France)) nucleic acid amplification for sequencing the haemagglutinin- (HA) and neuraminidase- (NA) gene was performed by reverse transcription using an iScript cDNA Synthesis Kit (Bio-Rad, CA, USA); amplification, purification, and sequencing was performed as previously described [19, 20]. Phylogenetic and molecular evolutionary analyses were performed using software package MEGA Version 4 (Kumar, Tamura, Nei, 2004). “Kimura-2” distance method and “Neighbour-Joining” algorithm were used for the phytogenic tree reconstruction.)

#### Influenza virus isolation and antigenic characterization

Influenza virus isolation was carried out in MDCK-SIAT1 cells (HPA Culture Collection, Cat.no. 05071502, passage 10) according to standard procedures [21]. Antigenic characterization of recovered influenza viruses was performed by haemaggluttination inhibition (HI) test, using reference viruses and postinfection ferret sera obtained from the Worldwide Influenza Centre, Francis Crick Institute, London according the protocols previously described [22]. Briefly, postinfection ferret antisera against the following reference viruses were used: A(H3N2): A/Texas/50/2012 and A/Switzerland/9715293/2013; A(H1N1)pdm09: A/California/7/2009; Influenza B: B/Massachusetts/02/2012, B/Phuket/3073/2013, and B/Brisbane/60/2008. HI test for influenza A(H3N2) viruses was carried out in the presence of 20nM Oseltamivir to address potential NA mediated binding to erythrocytes. Viruses were assigned as antigenically similar to the reference virus if the HI titre against the reference serum was ≤8-fold decreased compared to the homologous titre.

### Estimation of influenza vaccine effectiveness

**Fig 1:**
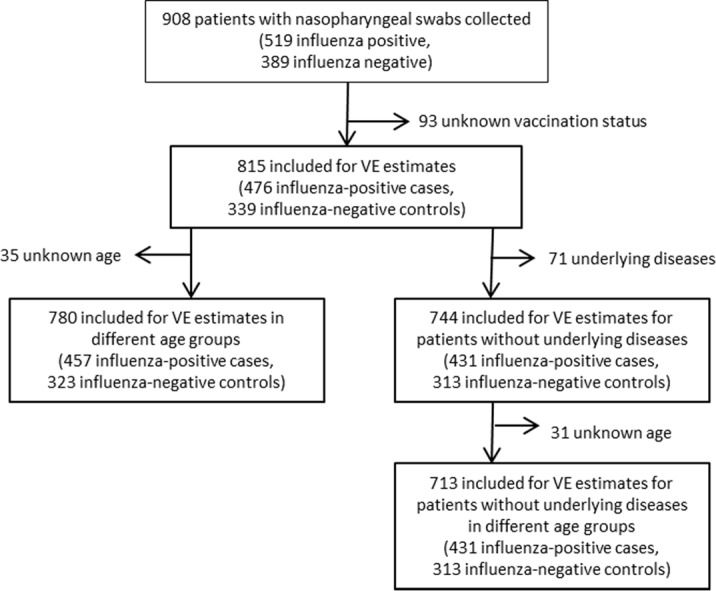
Inclusion and exclusion criteria. Inclusion and exclusion criteria applied to the data set used for VE estimates.

Inclusion criteria for this analysis were: specimen collection within 7 days after onset of ILI symptoms, and at least more than 2 weeks between vaccination and onset of ILI symptoms; further inclusion and exclusion criteria applied to the data set used for VE estimates are provided in Fig 1. Overall and component specific VE against laboratory confirmed influenza were estimated by use of the test-negative case-control design where a case is defined as a patient with influenza as confirmed by RT-PCR and a control is defined as a patient tested negative for influenza. Odds ratios (OR) for medically attended, laboratory-confirmed influenza were estimated by multivariate logistic regression applying three different models adjusting for different potential confounders. All models included gender as a covariate. For overall and subtype specific estimates also age was included into the model. The third model adjusted also for calendar week of onset of disease. Calculations were done using the generalized linear model with binomial counts and logit link (SPSS 23.0, IBM Corporation, USA).

The VE was calculated as (1-OR) x 100% to compare vaccination status of cases with controls. Crude and adjusted overall, age and subtype specific estimates of VE were calculated for all patients and stratified by underlying diseases. Age group specific estimates were limited by the low number of specimens obtained, especially from patients ≥65 years of age.

Frequencies of influenza types and subtypes detected in the different age groups were analysed using Fisher-Freeman-Halton Test (StatXact, Cytel Corp. USA). P-Values < 0.05 were considered significant.

## Results

### Influenza virus activity in Austria in the season 2014/15

Influenza virus activity started in week 2/2015, peaked in weeks 7 and 8 and ended in week 16/2015. Altogether 908 sentinel samples were tested for influenza viruses, of these 519 (57.2%) were influenza virus positive. Briefly, in 95 (18.3%) samples A(H1N1)pdm09, in 311 (59.9%) A(H3N2) and in 105 (20.2%) influenza B viruses were detected, further in 6 (1.2%) samples both influenza A subtypes were found. Due to the amount of material provided subtyping and further analysis of 2 (0.4%) influenza A samples was not possible.

In the course of the season a heterogeneous mix of circulating strains with changing dynamics and differing matches to the vaccine strains was observed.

**Fig 2:**
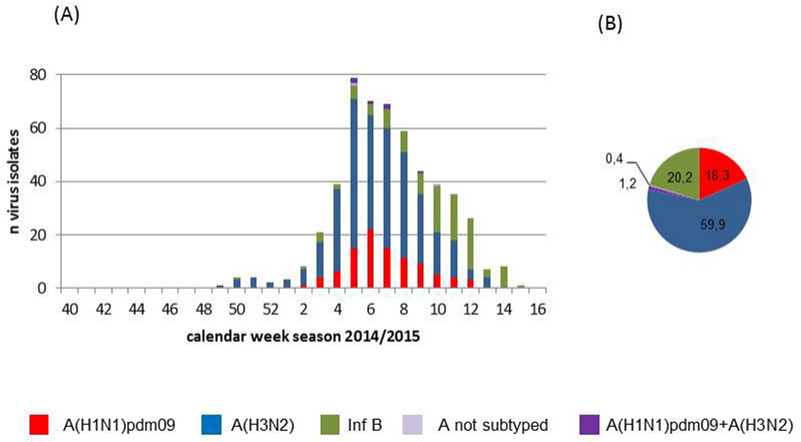
Influenza virus detection. Detection of influenza virus types and subtypes during the season 2014/15. (2A) number of influenza virus positive sentinel samples per week (2B) proportion of influenza virus types and subtypes (%) in sentinel samples.

At the beginning of the season A(H3N2) viruses dominated, representing 70% of the detected influenza viruses up to week 8/2015. Thereafter a continuous increase in the circulation of A(H1N1)pdm09 and Influenza B viruses was observed (Fig 2).

Influenza virus detection did not vary significantly (p = 0.548) by age group. The highest proportion of detected influenza viruses was 60% and was observed in the age group 0 to 14 years (with 156 of 262 samples influenza virus positive) and in the group 41 to 64 years (153 of 256 samples virus postive), followed by the age group of 15 to 20 years with 56% (37 of 66 samples virus positive). The proportion of detected influenza viruses in the 21 to 40 year old patients was 54% (134 of 247 samples virus positive). Only a low number of specimens were obtained from the elderly and in this group influenza viruses were detected in 50% (21 of the 42 samples virus positive). The patient’s age was not reported for 19 influenza patients.

**Fig 3:**
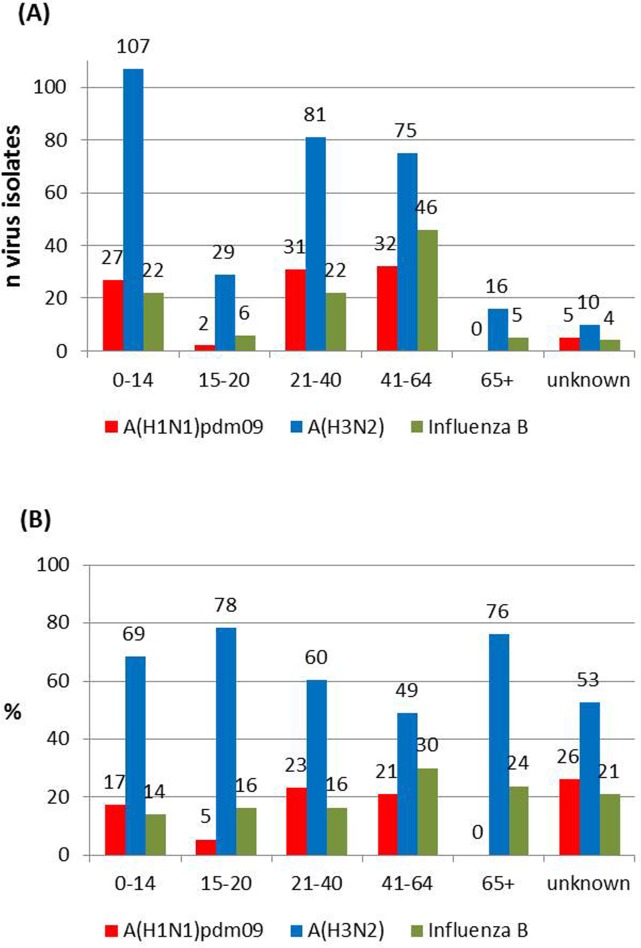
Influenza virus detections per age group. (3A) absolute numbers of influenza virus types/subtypes in the different age groups. (3B) proportion of influenza virus types/subtypes in % in different age groups.

A statistically significant difference (p = 0.002) was observed with regard to the frequency of influenza types and subtypes detected in the different age groups (see Fig 3A and 3B). A(H3N2) viruses accounted for the majority of influenza virus infections in all age groups, but the highest proportion of 78% was observed in the group of patients between 15 and 20 years of age, followed by the elderly with 76%. The proportion of detected A(H3N2) viruses in children was 69%. A lower proportion of detected A(H3N2) viruses was observed in adults aged between 21 and 40 years (60%) and between 41 and 64 years (49%).

Influenza A(H1N1)pdm09 viruses were responsible for 17% of the infections in children, but only for 5% in patients between 15 and 20 years of age. The proportion of detected A(H1N1)pdm09 viruses in adults aged between 21 and 40 and 41 and 64 years was quite similar with 23% and 21% respectively. A(H1N1)pdm09 viruses were not detected in elderly patients. Infections with influenza B viruses occurred in all age groups. The highest proportion of 30% was observed in adults between 41 and 64 years of age followed by the age group of elderly patients with 24% influenza B virus positive samples (Fig 3A and 3B).

### Genetic and antigenic characterisation of sentinel viruses

Out of the 519 influenza positive sentinel samples detailed genetic and antigenic analyses were performed for 98 A(H3N2), 59 A(H1N1)pdm09 and for 58 influenza B viruses.

**Fig 4:**
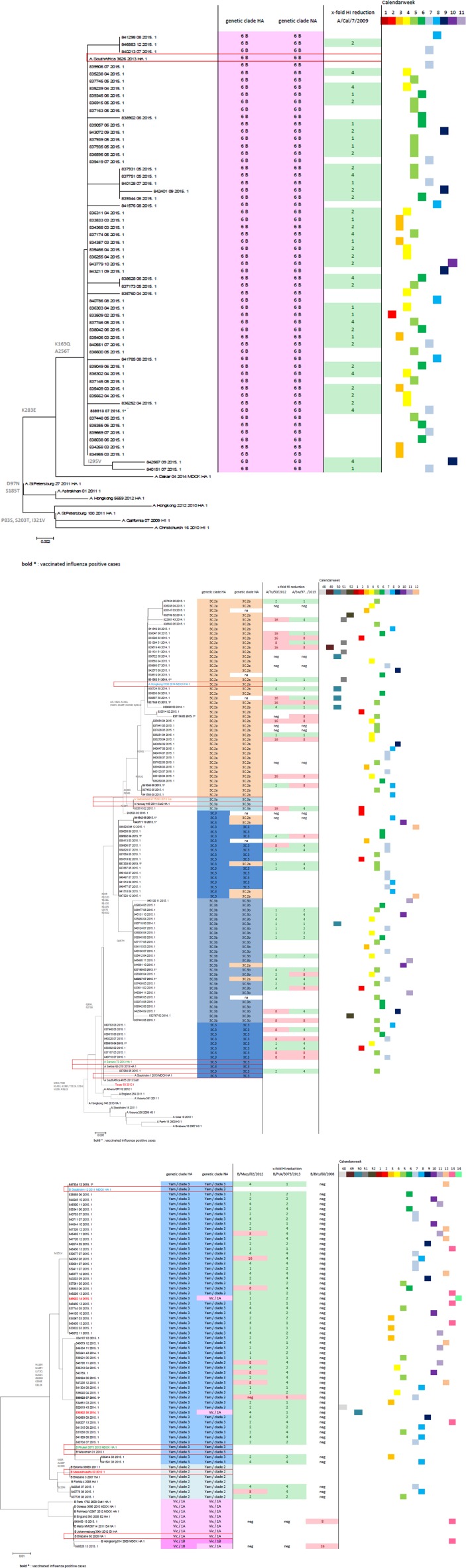
Phylogenetic analyses. Phylogenetic tree for the HA gene (AA based) as well as the results on the genetic analyses of the NA gene, antigenic typing results and information on the time of sample collection of each virus; viruses in colour and framed by red rectangles indicate reference viruses; viruses in bold and with * indicate vaccinated influenza positive cases; viruses in red and bold indicate influenza B viruses with HA and NA from distinct influenza B lineages. (4A) detailed results of 59 influenza A(H1N1)pdm09 viruses, (4B) detailed results of 98 A(H3N2) viruses, and (4C) detailed results of 58 influenza B viruses.

Genetic analyses of the HA and NA gene of the 98 A(H3N2) viruses revealed that 42 (43%) viruses belonged to the clade 3C.2a (representative of this clade A/Hong Kong/5738/2014), 28 (29%) clustered with clade 3C.3 viruses (represented by A/Samara/73/2013) whereas 5 of these viruses had an NA gene that belonged to clade 3C.2a. 27 (27%) were assigned to clade 3C.3b (representative A/Norway/0144/2015), but 2 of these had an NA gene that belonged genetically to clade 3C.2a. Further details are presented in Fig 4. Clade 3C.2a viruses comprised the majority of viruses analysed and differed in 8 to 9 amino acids at key antigenic sites of the HA gene from the vaccine strain. None of the 98 A(H3N2) viruses analysed belonged to the clade 3C.1, represented by the northern hemisphere 2014/15 vaccine strain A/Texas/50/2012 indicating substantial vaccine mismatch. Only one virus clustered with clade 3C.3a represented by A/Switzerland/9715293/2013, the recommended A(H3N2) vaccine strain for the upcoming season 2015/16. In contrast to the results obtained by sequencing, antigenic characterization indicated A/Switzerland/9715293/2013-like viruses in 57% of the A(H3N2) viruses analysed by HI assay.

Sequences of the HA and NA genes of the 59 A(H1N1)pdm09 viruses analysed clustered with clade 6B viruses, as represented by A/South Africa/3626/2013. In HI assay all 59 A(H1N1)pdm09 viruses were well recognized by the A/California/7/2009 post infection ferret serum indicating antigenic similarity with the vaccine strain.

Sequence analyses of the influenza B viruses showed that the majority of influenza B viruses were represented by lineage-matched B/Yamagata viruses with a clade-level variation. Briefly, 53 (91%) belonged to B/Yamagata clade 3 (represented by B/Phuket/3073/2013), but two viruses had an NA gene that belonged to B/Victoria clade 1A. Three viruses (5%) clustered within B/Yamagata clade 2 (represented by B/Massachusetts/02/2012), one virus was B/Victoria clade 1A, and one virus clustered with its HA gene in B/Victoria clade 1B and in its NA gene in B/Victoria clade 1A. Antigenic analysis revealed no significant difference to the B vaccine component.

The comparative analysis of the genetic and antigenic characterization results of viruses obtained from vaccinated and non-vaccinated patients revealed no differences between these two groups.

The phylogenetic tree for the HA genes of the influenza A(H1N1)pdm09, A(H3N2) and Influenza B viruses as well as the results of the genetic analyses of the respective NA genes, of antigenic typing results and information on the time of sample collection of each virus is provided in Fig 4.

### Vaccine effectiveness

Information about influenza vaccination was available from 815 of the 908 sentinel patients. The overall vaccination rate in our group of patients was 5.3%. The proportion of cases who had received influenza vaccine was 2.9%, and of the controls 8.6%. Among vaccinated patients only one had received LAIV for the season 2014/15 and was tested negative for influenza. The detailed information on influenza type/subtype, age group (using the age groups usually analysed in VE studies), gender, vaccination status and on the number of cases and controls as well as information on underlying diseases is provided in Table 1.

**Table 1:** Participants Profile.

	Distribution by case status n (%)			Vaccination coverage n (%) vaccinated within strata		
	overall	cases	controls	overall	cases	controls
**N (%)**	**815**	**476 (58)**	**339 (42)**	**43 (5)**	**14 (3)**	**29 (9)**
A(H1N1)pdm09		86 (18)	NA		1 (1)	NA
A(H3N2)		284 (60)	NA		11 (4)	NA
A(H1N1)pdm09 + A(H3N2)^a^		6 (1)	NA		0 (0)	NA
B		100 (21)	NA		2 (2)	NA
**Age group (years) (n = 775)**						
0–14	237 (31)	142 (32)	95 (30)	7 (3)	1 (1)	6 (6)
15–64	507 (65)	297 (68)	210 (66)	25 (5)	11 (4)	14 (7)
65 +	31 (4)	18 (4)	13 (4)	7 (22)	2 (11)	5 (38)
median (range)	31 (0–90)	31 (0–87)	29 (0–90)	NA	NA	NA
**Sex**						
Female	422 (52)	245 (51)	177 (52)	22 (5)	4 (2)	18 (10)
Male	393 (48)	231 (49)	162 (48)	21 (5)	10 (4)	11 (7)
**underlying diseases**						
No	744 (91)	431 (91)	313 (92)	35 (5)	13 (30)	22 (7)
Yes^b^	71 (9)	45 (9)	26 (8)	8 (11)	1 (16)	7 (27)

Profile of participants included in the vaccine effectiveness estimates calculations (n = 815; patient’s age was available for n = 775 participants), NA: not applicable

^a^ simultaneous detection of Influenza A(H1N1)pdm09 and A(H3N2)

^b^ underlying disease (n): coronary disease (13), hypertonia (17), COPD/Asthma (13), diabetes mellitus (17), cancer (3), immunosuppression (1), other: M. Parkinson, M. Bechterew, M. Raynaud, Polyarthritis, seizure disorder, Colitis ulcerosa,…(15)

Based on these data an overall crude unadjusted vaccine effectiveness (VE) of 68% was estimated for the season 2014/15. Crude component specific unadjusted VE estimates were 58% for A(H3N2), 88% for A(H1N1)pdm09 and 78% for influenza B viruses.

The overall vaccination rate in the group of patients with underlying diseases (n = 71, median age: 50.2 years, range 6 to 89 years) was 8.5%, with a vaccination rate of 2% in cases and of 27% in negative controls. Since only a single influenza case was found in a vaccinated patient of this group, which differed significantly with regard to the overall vaccination rate and the vaccination rate of the influenza negative control group from those without comorbidities all VE estimates that are presented hereafter were carried out stratified by underlying diseases.

Compared to the total group of patients lower VE estimates were observed after stratifying by underlying diseases: crude unadjusted overall VE estimates were 59%, with crude unadjusted VE estimates of 47% for A(H3N2), 84% for A(H1N1)pdm09, and 70% for influenza B virus. Crude and adjusted estimates of VE for the three different age-groups clearly indicated a decreasing VE with increasing age (Table 2). However age group specific estimates were limited by the low number of specimens obtained from patients ≥65 years of age (CI for crude VE estimates -428 to 93 and CI for VE estimates adjusted for sex and type/subtype -20 to 67).

**Table 2:** Vaccine effectiveness estimates.

		**Crude VE**^a^ **(95%CI)**	**VE**^b^ **(95%CI)**	**VE**^d^ **(95%CI)**
whole season	Total	59 (17 to 80)	61 (17 to 82)	70 (34 to 86)
age (years)	0–14	84 (-42 to 98)	86 (-27 to 99)	92 (17 to 99)
age (years)	15–64	43 (-32 to 76)	46 (-30 to 78)	54 (-13 to 82)
age (years)	65+	38 (-428 to 93)	37 (-20 to 67)	68 (-49 to 95)
		**crude VE**^a^ **(95%CI)**	**VE**^c^ **(95%CI)**	**VE**^e^ **(95%CI)**
whole season	A(H1N1)pdm09	84 (-17 to 98)	82 (-34 to 98)	88 (3 to 99)
	A(H3N2)	47 (-14 to 75)	53 (-9 to 80)	62 (8 to 84)
	B	70 (-28 to 93)	67 (-45 to 93)	67 (-45 to 93)
Week 40/14 to 8/15	All types	52 (-11 to 79)	49 (-26 to 79)	
Week 9 to 16/15	All types	72 (-7 to 93)	81 (6 to 96)	
Week 40/14 to 8/15	A(H1N1)pdm09	79 (-62 to 97)	75 (-98 to 97)	
Week 9 to 16/15	A(H1N1)pdm09	100	100	
Week 40/14 to 8/15	A(H3N2)	43 (-39 to 23)	42 (-54 to 78)	
Week 9 to 16/15	A(H3N2)	54 (-123 to 91)	75 (-16 to 95)	
Week 40/14 to 8/15	B	54 (-261 to 94)	46 (-36 to 79)	
Week 9 to 16/15	B	80 (-61 to 98)	81 (-61 to 98)	

Vaccine effectiveness estimates: stratified analysis–restricted to participants without underlying diseases (n = 744); (VE vaccine effectiveness, CI 95% confidence interval)

^a^ crude VE estimates (1-OR)x100

^b^ VE estimates calculated by multivariate logistic regression, adjusted for sex and type/subtype

^c^ VE estimates calculated by multivariate logistic regression, adjusted for sex and age

^d^ VE estimates calculated by multivariate logistic regression, adjusted for sex, type/subtype and calendar week

^e^ VE estimates calculated by multivariate logistic regression, adjusted for sex, age and calendar week

With adjustment for the covariates age and sex type/subtype specific VE estimates of 53% for A(H3N2), 82% for A(H1N1)pdm09 and 67% for influenza B virus were obtained (Table 2).

Since changes of the proportionate mix of strains with differing vaccine matches were observed over the season (see Fig 2), VE estimates were stratified by calendar week 40/2014 to 8/2015 and for week 9/2015 to 16/2015. As expected overall and component specific VE estimates differed significantly between these two time periods of the influenza season (see Table 2). In addition VE was also estimated adjusted by multivariable logistic regression for calendar week of influenza virus infection. The highest VE estimates were obtained after full adjustment for all covariates, whereby the calendar week of infection was the covariate exerting the highest influence on adjusted VE estimates (Table 2).

Data on prior immunisation were available from 687 of the 744 patients without comorbidities. Of those 647 (94%) had not received the influenza vaccine for the previous season 2013/14.

## Discussion

Influenza virus evolution is a dynamic phenomenon that affects the level of vaccine induced protection each season. This paper presents data obtained by the Austrian sentinel surveillance system on the evolution of influenza viruses during the season 2014/15 and its impact on influenza vaccine effectiveness in primary care in Austria as estimated by a test-negative case control design. This design to estimate VE has been proven useful by many studies to provide the most reliable data for influenza VE, especially when highly sensitive and specific tests for laboratory confirmation of influenza like realtime RT-PCR methods are used [23–25].

The heterogeneous mix of circulating influenza virus strains of the different types and subtypes with differing matches to the vaccine strains observed over the season 2014/15 stresses the importance to perform VE estimates in the context of detailed characterization data of the contributing viruses. For influenza virus surveillance both, antigenic and genetic characterization of circulating viruses were performed in order to assess the appearance of relevant drift variants. The results of the study clearly demonstrate that antigenic characterization using the conventional HI assay failed to detect the relevant A(H3N2) drift variants in time, mostly due to the unavailability of antisera during the season against newly evolving viruses. Genetic characterization revealed from the very beginning of the epidemic influenza virus activity in the season 2014/15 the dominant circulation of influenza A(H3N2) viruses with a profound mismatch to the A(H3N2) vaccine strain accounting for 70% of the influenza cases up to week 8/2015. As expected the crude unadjusted VE stratified by underlying diseases against influenza for the first weeks of the season was only 52% and against circulating A(H3N2) viruses as low as 43%. Similar low interim estimates of 2014/15 VE against influenza A(H3N2) viruses have also been reported from Canada’s sentinel physicians surveillance network for those who received the 2014/15 vaccine without prior vaccination in 2013/14 [26]. These results are in agreement with our findings, as 94% of our sentinel patients had not received the 2013/14 vaccine. Since prior immunisation with an identical antigenic vaccine component may have a negative effect on the immune response [27, 28], extremely low interim VE estimates (-15%) in Canada were obtained for those with prior vaccination in 2013/14 [26]. A very low mid- and end-season effectiveness in preventing A(H3N2) infection has also been reported from the UK (-2,3%) [29, 30]; whereby information on the history of prior vaccination is not provided in these reports. In contrast to A(H3N2) the component specific crude VE stratified by underlying diseases for A(H1N1)pdm09 viruses and influenza B viruses with a good match to the respective vaccine strains were as high as 84% and 70% for the whole season.

Since a continuously changing pattern of circulating influenza strains with differing matches to the vaccine strains was observed over the whole season, the calendar week of influenza virus infection was the factor that influenced VE estimates most (Table 2). This was evident when VE estimates were stratified by week 40/2014 to 8/2015 and week 9/2015 to 16/2015 and resulted in significantly higher VE estimates after adjustment for this covariate.

In addition to the calendar week of infection, the calendar week of immunization is another aspect that may influence influenza VE. A lower influenza VE at the end of the season has been reported in an European multicentre case- control study and waning immunity, besides virus changes, was suggested as a possible explanation for this finding [15]. In this study VE estimates were not presented in the context of detailed genetic and antigenic analyses of the circulating influenza virus strains. In our sentinel study an increase of influenza VE over the season was observed and the higher VE in the second part of the season correlated with the increased circulation of influenza virus strains with a better match to the vaccine strains. As the 2014/15 influenza virus activity in Austria lasted for a period of 16 weeks, and HAI and NAI titers following vaccination decrease slowly over a period of 18 months [31], it seems unlikely that the calendar week of immunization exerts a great influence on VE estimates in our study.

Vaccine performance is not only influenced by the virus characteristics, but also by different factors like the patient’s age, underlying diseases, and the patient’s immune system which is influenced by the individual history of infections and/or vaccinations [27, 28, 32]. In order to consider some of these influencing factors VE estimates were carried out adjusted for age and stratified by underlying diseases.

In contrast to previously published studies on influenza VE stratifying by underlying diseases resulted in a decrease of overall VE estimates in our group of patients without comorbidities. This may be due to several factors: First of all, our group of patients with underlying diseases and with a median age of 50 years was relatively young and the proportion of immunosuppressed patients in this group was as low as 5.6% (one immunosuppressed patient and 3 patients with cancer). Secondly, for this group of patients annual influenza vaccination is highly recommended resulting in a higher vaccination rate with only one laboratory confirmed influenza case in a vaccinated patient of this group. In addition patients with comorbidities suffering from respiratory infections may differ in their healthcare-seeking behaviour and therefore respiratory samples may have been collected more frequently from this group of patients, resulting in inflated VE estimates. Besides, it also has to be considered that patients with underlying diseases living in the community have in general a similar risk to acquire influenza virus infection as the rest of the population. The important difference observed in these patients is the increased risk of a severe clinical course of influenza frequently requiring hospitalisation. Therefore, more reliable influenza VE estimates for this group of patients may be obtained by also assessing VE against influenza related hospitalisation [33].

An additional bias that affects VE estimates are differences in influenza attack rates and in viral shedding in different age groups in the course of influenza disease. Lower influenza attack rates are often observed in older people due to the presence of broadly cross-reactive antibodies resulting from previous influenza virus contacts [34, 35]. In addition, elderly patients shed virus at lower concentrations and for a shorter period of time and tend to present for care later in the course of illness compared to children and young adults which results in false negative laboratory test results. Also in our study VE estimates are based mostly on healthy children and adults and our sentinel population included only few elderly persons. This limitation of our study tends to be a general weakness of published VE calculations and therefore more studies related to an elderly sentinel population are needed to acquire more reliable data on VE estimates in the older age groups.

Our study, like all other case control studies, is predicated on the assumption that vaccinated persons have the same likelihood of being exposed to influenza as non-vaccinated persons, and that vaccinated and unvaccinated have the same healthcare-seeking behaviour, and that sampling of respiratory specimens is performed with equal frequency in both groups [36].

Another limitation to the surveillance approach is the sample size needed to allow analysis to be stratified by potentially important factors like age, influenza type/subtype, and underlying diseases. This is also the case in our study, where sample sizes are relatively small especially in the vaccinated group, due to the extremely low vaccination rate 2014/15 in Austria. Nevertheless the VE estimates obtained in our study compare quite well to those published for the season 2014/15 [26, 29].

Altogether the VE data described in this study underscore the importance to perform VE estimates in the context of detailed genetic and antigenic characterization of the contributing viruses and also shows that the calendar week of influenza virus infection is the most important confounder for VE estimates especially in seasons with a changing dynamic in circulating types/subtypes and strains. These data may contribute to the understanding of the impact of virus variations on VE estimates.

## Supporting Information

S1 File GISAID-Sequence-Accession-Numbers. Sequences of the haemagglutinin and/or the neuraminidase gene contributing to the 2014/15 influenza vaccine effectiveness analysis were deposited in GISAID with accession numbers provided in this file.
(DOCX)

S2 File Vaccine effectiveness calculation raw data. Raw data used for vaccine effectiveness estimates are provided in the four excel-sheets according to participants with and without underlying diseases, gender and vaccination status during the seasons 2014/15 and 2013/14.
(XLSX)
