# Supplementary material for: Detailed Report on 2014/15 Influenza Virus Characteristics, and Estimates on Influenza Virus Vaccine Effectiveness from Austria’s Sentinel Physician Surveillance Network
Source: PLoS One. 2016 Mar 14;11(3):e0149916. doi: 10.1371/journal.pone.0149916 (PMC4790898; doi:10.1371/journal.pone.0149916)
Supplement: S1 File — Sequences of the haemagglutinin and/or the neuraminidase gene contributing to the 2014/15 influenza vaccine effectiveness analysis were deposited in GISAID with accession numbers provided in this file. (DOCX) [file pone.0149916.s001.docx]

S1 File:

GISAID-Sequence-Accession-Numbers

Viruses from sentinel specimen with complete or partial sequences of the haemagglutinin and/or the neuraminidase gene contributing to the 2014/15 influenza vaccine effectiveness analysis were deposited in GISAID with accession numbers: EPI553473- EPI553482, EPI554626- EPI554637, EPI555141- EPI555148, EPI556498- EPI556503, EPI556885, EPI556886, EPI556895, EPI556896, EPI557516, EPI557518- EPI557523, EPI557525- EPI557532, EPI559421- EPI559434, EPI559463- EPI559473, EPI562304- EPI562321, EPI566396- EPI566423, EPI566433- EPI566436, EPI566440- EPI566443, EPI566445- EPI566448, EPI566689- EPI566706, EPI566771- EPI56676, EPI566910- EPI566923, EPI567821- EPI567840, EPI567918- EPI567925, EPI569327- EPI569359, EPI572697- EPI572710, EPI573144, EPI573145, EPI576149, EPI573150, EPI573156, EPI573157, EPI573685- EPI573704, EPI576445- EPI576467, EPI579941- EPI579952, EPI579954, EPI576955, EPI579957, EPI576958, EPI580302, EPI580303, EPI580306- EPI580309, EPI580314, EPI580316, EPI580319, EPI580320, EPI580322, EPI580323, EPI580332, EPI580333, EPI580341, EPI580343, EPI582280- EPI582289, EPI582296- EPI582313, EPI582315, EPI583951- EPI583968, EPI584001- EPI584017, EPI584096, EPI584101- EPI584110, EPI584113, EPI584114, EPI854121, EPI584123
